# Catalyzing IVF outcome prediction: exploring advanced machine learning paradigms for enhanced success rate prognostication

**DOI:** 10.3389/frai.2024.1392611

**Published:** 2024-11-05

**Authors:** Seyed-Ali Sadegh-Zadeh, Sanaz Khanjani, Shima Javanmardi, Bita Bayat, Zahra Naderi, Amir M. Hajiyavand

**Affiliations:** ^1^Department of Computing, School of Digital, Technologies and Arts, Staffordshire University, Stoke-on-Trent, United Kingdom; ^2^Department of Computer Engineering, Razi University, Kermanshah, Iran; ^3^Leiden Institute of Advanced Computer Science, Leiden University, Leiden, Netherlands; ^4^Department of Computer Engineering, Artificial Intelligence, Islamic Azad University, Malard, Iran; ^5^Obstetrics and Gynaecology Department, Iran University of Medical Sciences, Tehran, Iran; ^6^Department of Mechanical Engineering, School of Engineering, University of Birmingham, Birmingham, United Kingdom

**Keywords:** *in vitro* fertilization, predictive modeling, machine learning, feature engineering, data preprocessing, hyperparameter tuning, algorithm selection, feature selection

## Abstract

This study addresses the research problem of enhancing *In-Vitro* Fertilization (IVF) success rate prediction by integrating advanced machine learning paradigms with gynecological expertise. The methodology involves the analysis of comprehensive datasets from 2017 to 2018 and 2010–2016. Machine learning models, including Logistic Regression, Gaussian NB, SVM, MLP, KNN, and ensemble models like Random Forest, AdaBoost, Logit Boost, RUS Boost, and RSM, were employed. Key findings reveal the significance of patient demographics, infertility factors, and treatment protocols in IVF success prediction. Notably, ensemble learning methods demonstrated high accuracy, with Logit Boost achieving an accuracy of 96.35%. The implications of this research span clinical decision support, patient counseling, and data preprocessing techniques, highlighting the potential for personalized IVF treatments and continuous monitoring. The study underscores the importance of collaboration between gynecologists and data scientists to optimize IVF outcomes. Prospective studies and external validation are suggested as future directions, promising to further revolutionize fertility treatments and offer hope to couples facing infertility challenges.

## 1 Introduction

In recent times, *In-vitro* fertilization (IVF) has emerged as a popular solution for addressing complications associated with infertility (Cascante et al., 2023). Infertility affects more than 80 million couples worldwide, prompting the need for effective reproductive interventions (Zafar et al., 2023). IVF offers a promising pathway to overcome various challenges like endometriosis, genetic disorders, poor egg quality, and sperm-related issues (Wu et al., 2022). While IVF provides hope to many, it comes with significant uncertainties, including high costs and variable success rates (Franklin, 2022). This paper delves into the application of advanced machine learning paradigms to predict IVF success rates, aiming to provide a more accurate prognosis for couples undergoing the procedure.

IVF, a groundbreaking assisted reproductive technology, involves fertilizing eggs with sperm outside the body and then transferring the resulting embryos to the woman’s uterus (Calandrillo and Deliganis, 2015). This technique has led to the birth of over 5 million babies globally, providing a viable solution for individuals grappling with fertility challenges (Adamson et al., 2013). The process is multifaceted, encompassing medical procedures, surgeries, and multiple cycles, often spanning several months before achieving a successful pregnancy. Despite its potential benefits, the decision to pursue IVF is complicated by factors such as high costs, uncertainty, and emotional stress. These factors contribute to patients discontinuing treatment prematurely due to the physical and psychological burden (Hammarberg et al., 2001).

Given the intricate nature of IVF and the associated challenges, accurate prediction of success rates becomes paramount. Traditional methods of prediction are often subjective, relying heavily on the experience of individual medical practitioners. This limitation necessitates the development of systematic and statistically driven approaches that can provide reliable predictions. This is where Artificial Intelligence (AI) and Machine Learning (ML) step in, offering data-driven solutions that can transform decision-making processes in healthcare, including IVF.

The core problem addressed in this study is the unpredictability of IVF outcomes and the need for a more accurate prediction model. While various prediction models exist, they often lack comprehensive evaluation across different machine learning paradigms (Iftikhar et al., 2020). The primary objective of this research is to explore and compare advanced machine learning algorithms to predict live-birth occurrences in IVF cycles. The study focuses specifically on cases where embryos are formed from couples’ gametes, rather than donor gametes.

Machine Learning, a branch of AI, equips computers with the ability to learn from past experiences and make predictions (Sadegh-Zadeh et al., 2023; Surden, 2014; Carson and Kallen, 2021; Sadegh-Zadeh et al., 2024). It offers a powerful means of analyzing complex patterns in data, resembling human decision-making processes. Neural network, a subset of ML, simulates human neural networks to identify intricate patterns that might elude human analysis (Aggarwal, 2018; Nazari et al., 2024; Sadegh-Zadeh et al., 2023). In healthcare, ML and DL have already demonstrated their efficacy in personalized care, drug discovery, disease diagnosis, and surgery simulations. Applying these techniques to reproductive science holds the potential to enhance IVF success prediction (Louis et al., 2021; Sadegh-Zadeh et al., 2019).

This paper is structured to provide a comprehensive exploration of the application of advanced machine learning paradigms to IVF success prediction. The subsequent sections detail the methodology employed, including data collection and preprocessing techniques. It also outlines the various machine learning models utilized in the study. The results and discussion section presents a thorough comparison of model performance, using metrics such as F1-score, precision, recall, and ROC-AUC curves. By analyzing these metrics, the most effective model for IVF success prediction is determined.

## 2 Related work

The field of reproductive medicine, particularly *in vitro* fertilization (IVF), has seen substantial advancements in recent years. These advancements have been coupled with the integration of machine learning techniques, creating a dynamic landscape where predictive models are being developed to enhance the efficiency and success rates of IVF treatments (Katler et al., 2022). This literature review aims to provide an overview of the existing research in two key areas: IVF success prediction and the application of machine learning in healthcare, while also identifying the gaps that our study seeks to address.

### 2.1 *In-vitro* fertilization success prediction

In recent years, the field of assisted reproductive technology (ART) has witnessed substantial advancements, especially in the realm of *in vitro* fertilization (IVF) and intracytoplasmic sperm injection (ICSI) (Feuer and Rinaudo, 2016). As IVF and ICSI have become increasingly prevalent treatments for infertility, researchers and clinicians have turned to various methodologies, including machine learning and artificial intelligence, to predict treatment outcomes, improve success rates, and optimize patient care (Siristatidis et al., 2021).

In the study conducted by Smeenk et al. (2005), the intricate relationship between psychological factors, stress hormones, and the outcomes of IVF/ICSI treatments was examined. The research involved collecting nocturnal urine samples from women undergoing their first IVF/ICSI cycle and assessing their stress hormone concentrations along with administering anxiety and depression questionnaires before treatment initiation. The findings revealed a notable positive correlation between urinary adrenaline concentrations at baseline and embryo transfer (ET) and depression scores at baseline. Additionally, successful treatment outcomes were associated with lower levels of adrenaline at oocyte retrieval and lower levels of both adrenaline and noradrenaline at ET, compared to unsuccessful treatments. These results underscored the significance of stress hormones, particularly adrenaline, in the intricate relationship between psychosocial stress, emotional well-being, and the outcomes of IVF/ICSI treatments.

Abdulrahim et al. (2021) investigated couples’ preferences regarding fresh embryo transfer versus freezing of all embryos and their associated clinical outcomes in IVF treatments. The study found that couples’ preferences were primarily influenced by factors such as the chances of live birth, miscarriage, neonatal complications, and treatment costs, rather than differences in the treatment process, including the delay in embryo transfer with frozen embryos or the risk of ovarian hyperstimulation syndrome (OHSS) associated with fresh embryo transfer. The study utilized a discrete choice experiment (DCE) to survey infertile couples and revealed that they favored IVF techniques that offered higher live birth rates and lower rates of miscarriage and neonatal complications. The findings highlighted the importance of balancing success and safety in IVF treatments and emphasized the need to consider patient preferences for expected clinical outcomes and risks in individualized care.

Henderson et al. (2021) introduced an innovative counseling tool that utilizes predictive models to estimate the likelihood of live birth prior to IVF treatment. This tool integrates pre-treatment variables such as maternal age, ovarian reserve, and treatment history to offer patients informed expectations. Using logistic regression, the study identifies crucial predictors that notably impact IVF success rates. By furnishing prospective parents with a personalized prediction tool, this research enables patients to make well-informed choices regarding their fertility treatment path, contributing to the broader effort of incorporating predictive models into clinical practice for assisted reproductive technology treatments.

Hassan et al. (2020) conducted a study illustrating the remarkable predictive capabilities of machine learning in the context of IVF outcomes. Utilizing a range of machine learning techniques, including classic algorithms, neural network, and ensemble methods, they analyzed the Human Fertilization and Embryology Authority (HFEA) dataset to predict live births following IVF/ICSI treatment. Their research demonstrated that machine learning models could achieve accuracy rates of up to 76.49% when predicting treatment outcomes, underlining the potential and practical relevance of machine learning in clinical settings.

Majumdar et al. (2023) addressed the pressing issue of personalized prediction within the realm of initial IVF cycles, aiming to alleviate the socioeconomic stress associated with infertility. Their study leveraged a dataset comprising 2,268 patients who underwent IVF/ICSI procedures, spanning from January 2018 to December 2020 at the Center of IVF and Human Reproduction, Sir Ganga Ram Hospital. With 79 relevant features encompassing factors such as maternal age, IVF cycle count, infertility type, duration, AMH levels, indication for IVF, sperm type, BMI, embryo transfer details, and *β*-hCG values, a machine learning model was meticulously constructed. The deep Inception-Residual Network architecture-based neural network stood out as the most promising classifier, achieving an impressive accuracy rate of 76% and an ROC-AUC score of 0.80, surpassing other models. This groundbreaking approach, pioneering in the realm of reproductive health, provides a novel avenue for delivering personalized predictions of IVF success, equipping both clinicians and patients with valuable insights to make informed decisions regarding their fertility journey.

Simopoulou et al. (2018) conducted a groundbreaking study aimed at optimizing IVF treatment outcomes by incorporating omics data and artificial intelligence. Their research involved the integration of personal and lifestyle information from various European populations to construct a comprehensive model for treatment recommendations. Utilizing advanced mathematical methods, such as neural networks, the researchers sought to identify critical factors influencing successful IVF outcomes. This innovative approach highlighted the capacity of AI to harness intricate datasets and provide valuable insights for tailoring personalized treatment strategies in line with the evolution of assisted reproductive technology, ultimately contributing to safer, more effective IVF treatments.

In an extensive prospective observational cohort study conducted at a university-affiliated private infertility center, Vaegter et al. (2017) developed a predictive model for live birth outcomes following *in vitro* fertilization/intracytoplasmic sperm injection (IVF/ICSI) treatments, specifically focusing on single-embryo transfer (SET) after 2 days of embryo culture. Their analysis of 8,451 IVF/ICSI treatments involving 5,699 couples over a 15-year period revealed seven independent predictors of live birth: embryo score, ovarian sensitivity index (OSI), female age, treatment history, endometrial thickness, infertility cause, and female height. These factors collectively contributed to the construction of a prediction model with moderate discrimination but high accuracy in subgroups of patients, offering live birth rate (LBR) estimates ranging from <10 to >40%. The study’s internal validation data set, comprising 2,460 cases, confirmed the model’s excellent calibration. Notably, this research marked the first inclusion of female height as a predictor of live birth after IVF/ICSI, presenting a valuable tool for guiding medical professionals and prospective parents in their decision-making processes.

In recent years, time-lapse imaging has emerged as a powerful tool for improving IVF outcome prediction by continuously monitoring embryo development. Borna et al. (2024) introduced a predictive model that utilizes time-lapse images captured at different stages of embryo development to predict pregnancy outcomes. Their approach demonstrated improved prediction accuracy by leveraging dynamic embryo characteristics that are not visible in static images. Similarly, Sawada et al. (2021) explored the use of time-lapse imaging combined with deep learning techniques to predict live birth outcomes, further highlighting the potential of this technology to enhance IVF success predictions. These studies demonstrate the value of continuous embryo monitoring in refining prediction models, offering richer data for analysis compared to traditional methods.

### 2.2 Application of machine learning in healthcare

The integration of machine learning in healthcare has shown immense promise, with applications spanning diagnosis, treatment planning, drug discovery, and patient management. In the field of reproductive medicine, a recent study by Chavez-Badiola et al. (2020) exemplifies this trend by addressing a longstanding challenge in assessing the viability of blastocysts for pregnancy prediction. Leveraging artificial vision and machine learning techniques, the researchers developed a novel algorithm that predicts pregnancy outcomes using the beta human chorionic gonadotropin (b-hCG) test, combining information from the morphology of embryos and the age of patients. The algorithm was trained and evaluated on two high-quality databases with known pregnancy outcomes (*n* = 221) and utilized multiple classifiers, including probabilistic Bayesian, Support Vector Machines (SVM), neural networks, decision trees, and Random Forest (RF). Results from their study demonstrated promising predictive capabilities, with SVM achieving an F1 score of 0.74 and an AUC of 0.77 in one database, and RF obtaining an F1 score of 0.71 and an AUC of 0.75 in another. This research offers a significant advancement in the field, as it presents a system capable of predicting positive pregnancy test outcomes from a single digital image, offering a practical and adaptable approach for clinical settings, with potential implications for improving reproductive healthcare outcomes.

While several studies have explored predictive models for IVF success, significant research gaps remain:

1. Limited integration of diverse factors: Existing models often rely on a narrow set of features, overlooking the potential benefits of incorporating a broader range of clinical, demographic, and procedural factors. Our study addresses this by using a comprehensive dataset that includes 40 variables, providing a more holistic analysis of IVF outcomes.
2. Underutilization of advanced machine learning techniques: Although some models have used basic machine learning algorithms, there is a lack of exploration of more advanced ensemble methods (e.g., AdaBoost and LogitBoost) which can improve prediction accuracy. Our work fills this gap by comparing various ensemble learning models.
3. Inadequate handling of missing data: Most studies either omit records with missing data or use simple imputation methods, which can introduce bias. We propose a novel approach to missing data handling that retains potentially valuable information and mitigates the risk of overfitting.
4. Lack of longitudinal validation: Existing models often fail to test their predictive capabilities across multiple time periods, raising concerns about their generalizability. Our study evaluates model performance across two datasets from different timeframes (2010–2016 and 2017–2018), demonstrating robustness and consistency.
5. Limited clinical interpretability: Many machine learning models lack the interpretability needed for clinical application, which limits their usefulness in real-world decision-making. We address this by involving gynecologists and ensuring that the model outputs are clinically meaningful and actionable.

By addressing these specific research gaps, our study contributes to advancing the field of IVF outcome prediction. One of the key research gaps we identified is the lack of longitudinal validation in previous studies. While our study uses data spanning time periods (2010–2016 and 2017–2018), the consistency in patient demographics, treatment protocols, and clinical variables across these periods allows us to ensure that the model can generalize across different timeframes. Although there were minimal changes in IVF procedures during these years, the inclusion of both datasets serves to enhance the model’s robustness, confirming that it can predict outcomes accurately over different time spans.

### 2.3 Our contribution

In light of the existing research landscape, our study aims to address these gaps by developing a comprehensive IVF success prediction model that leverages a diverse set of pre-treatment parameters. By integrating a broader spectrum of factors such as clinical, demographic, and omics data, our model seeks to provide more accurate and personalized predictions. By integrating clinical, demographic, procedural, and omics data, our model provides more accurate and personalized predictions for IVF success. Clinical data, such as patient age, hormone levels, and causes of infertility, capture key reproductive health metrics, while demographic factors, like patient and partner ethnicity, account for genetic and lifestyle influences. Procedural data, including the use of fresh or frozen embryos and whether eggs or sperm were donor-sourced, add precision to the model by reflecting specific treatment protocols. Although omics data is still emerging in IVF, our model is designed to incorporate it in the future, offering the potential for even deeper insights into the molecular factors affecting fertility. This comprehensive approach enhances the model’s predictive accuracy and utility in clinical decision-making. Furthermore, we aim to enhance the interpretability of the prediction process by employing advanced machine learning techniques, enabling healthcare professionals to make informed decisions based on the model’s outputs.

Our contributions can be summarized as follows:

- Integration of a broader spectrum of features: We incorporated clinical, demographic, and procedural factors, along with the potential for integrating omics data in future models, to provide more personalized IVF outcome predictions.
- Application of advanced ensemble learning techniques: Our work explores and compares various advanced machine learning models, including AdaBoost and LogitBoost, to improve prediction accuracy.
- Novel handling of missing data: We developed an innovative method for dealing with missing data by using numerical placeholders, preserving the integrity of the dataset while minimizing bias.
- Evaluation across multiple datasets: Our models were tested on two datasets from different time periods (2010–2016 and 2017–2018), demonstrating consistency and robustness.
- Clinical interpretability: We emphasized the importance of developing models that are interpretable by healthcare professionals, enabling actionable insights for decision-making in IVF treatments.

The literature review underscores the progress made in IVF success prediction and the application of machine learning in healthcare. However, it also highlights the need for more inclusive and refined prediction models that consider a wider array of parameters. Our study seeks to contribute to this field by developing a robust prediction model that could potentially revolutionize the way IVF treatments are planned and executed, leading to improved success rates and enhanced patient outcomes.

Numerous studies have explored IVF outcome prediction using machine learning techniques, but the reported accuracy varies widely. For example, Simopoulou et al. (2018) reported accuracy rates ranging from 57 to 76% across different models, while Hassan et al. (2020) achieved a maximum accuracy of 91% using advanced neural network techniques. However, the predictive performance of these models still leaves room for improvement. In our study, ensemble models like AdaBoost and LogitBoost achieved a maximum accuracy of 96.35%, representing a significant improvement of approximately 5.35% over the highest previously reported accuracy. This improvement underscores the effectiveness of our approach, which integrates a broader set of clinical and demographic features and employs advanced machine learning methods to enhance the robustness and accuracy of predictions.

## 3 Data set description and preprocessing

### 3.1 Dataset

The 2010–2016 dataset is an anonymized registry from the Human Fertilization and Embryology Authority, covering fertility treatments from 2010 to 2016. It represents one of the most extensive and longest-running fertility treatment registries worldwide, aiming to improve patient care while ensuring the confidentiality of patients, donors, and offspring. The dataset contains 495,630 patient records, with 94 features describing various aspects of fertility treatment cycles, including patient demographics, treatment types, and detailed causes of infertility, providing a comprehensive view of each treatment cycle during this period.

The dataset features both numerical and categorical data. Key attributes include the patient’s age at treatment, number of IVF pregnancies, live births, and specific causes of infertility, such as tubal disease, ovulatory disorder, and male factors. Additionally, it captures details about the type of eggs and sperm used in treatments (e.g., fresh or frozen, donor or patient), the number of eggs collected, and the number of embryos transferred during each cycle.

In addition to the 2010–2016 dataset, we utilized a separate 2017–2018 dataset of fertility treatment records to evaluate our model. These datasets are not simply split versions of a single source but represent different time periods. Evaluating across both datasets allowed us to verify model performance consistency and robustness over different temporal spans, ultimately reinforcing the reliability of our findings.

Our study expands on previous research by introducing a broader and more comprehensive set of parameters for IVF outcome prediction. While prior studies have predominantly focused on patient age, number of IVF cycles, and basic clinical parameters, we have included a more diverse set of 40 features covering clinical, demographic, and procedural factors. Key parameters newly considered in our research include:

1. Embryo-related data: Detailed information on the number of embryos thawed, transferred, and stored.
2. Infertility causes: Expanded categories of infertility causes, such as endometriosis and unexplained infertility.
3. Ethnicity and demographic factors: Patient and partner ethnicity, which were not widely considered in previous models.
4. Treatment details: Information on treatment methods like elective single embryo transfer and specific hormonal stimulation approaches.
5. Egg and sperm source: Data on whether eggs or sperm were sourced from a donor or the patient, adding more precision to outcome predictions.

By considering these newly introduced parameters, our model provides a more holistic and accurate prediction of IVF success, addressing limitations found in previous research.

The preprocessing phase encompassed several critical steps. One such step involved the application of a normalization technique, which served to standardize the scale of all feature values. To ensure uniform scale among numerical features, a Standard Scaler was applied, thereby standardizing the dataset. This step is crucial, particularly for algorithms that exhibit sensitivity to variations in feature magnitudes. The dataset itself exhibits a combination of string and numeric data types, reflecting the diverse nature of the features collected. The ensuing enumeration presents a catalog of the features encompassed within the dataset which have been presented in Table 1. It details various parameters such as patient age at treatment, the total number of previous IVF and DI cycles, and a range of causes for infertility, including tubal disease, ovulatory disorder, and male factors. Additionally, it lists factors related to the embryos, such as the number of embryos transferred, frozen, and thawed, as well as outcomes like live birth occurrences and early outcomes. The Table 1 also covers demographic and procedural variables, including donor age, patient and partner ethnicity, and type of treatment. This comprehensive list indicates a multifaceted approach to analyzing fertility treatments, aiming to understand the numerous factors that could influence the success rates of IVF and DI procedures.

**Table 1:** Description of 37 fields in the dataset.

Feature	Description
1. Patient age	Age of the patient at the time of treatment
2. Number of embryos transferred	Total embryos transferred during the procedure
3. Infertility cause	The underlying cause of infertility
4. Embryo quality	Quality grading of the embryos
5. Hormonal profile	Hormonal levels measured prior to IVF treatment
6. Stimulation protocol	Type of stimulation protocol used
7. Previous IVF cycles	Number of previous IVF cycles attempted
8. Partner’s age	Age of the patient’s partner
9. Body mass index (BMI)	Body mass index of the patient
10. Treatment type	Whether the treatment was fresh or frozen
11. Hormonal medications	Type of medications used for ovarian stimulation
12. Duration of infertility	Number of years the couple has experienced infertility
13. Tubal factor	Presence of tubal factors affecting infertility
14. Ovulatory factor	Presence of ovulatory dysfunction
15. Male factor	Presence of male factor infertility
16. Unexplained infertility	Cases where infertility cannot be explained
17. Endometrial thickness	Endometrial thickness measured prior to transfer
18. Embryo stage	Stage of embryo development at transfer
19. Number of eggs retrieved	Total eggs retrieved during the cycle
20. Gonadotropin dosage	Dosage of gonadotropins used in stimulation
21. Fertilization method	IVF or ICSI procedure
22. Embryo freezing	Whether any embryos were frozen
23. Partner’s smoking status	Smoking status of the patient’s partner
24. Patient’s smoking status	Smoking status of the patient
25. Alcohol consumption	Whether the patient consumes alcohol
26. Patient ethnicity	Ethnicity of the patient
27. Partner ethnicity	Ethnicity of the patient’s partner
28. Patient occupation	Patient’s occupation
29. Partner occupation	Partner’s occupation
30. Socioeconomic status	Socioeconomic background of the patient
31. Genetic screening	Whether genetic screening was conducted
32. Uterine abnormalities	Presence of any uterine abnormalities
33. Endometriosis	Diagnosis of endometriosis
34. Fertility preservation	Whether the patient is undergoing fertility preservation
35. Presence of fibroids	Presence of fibroids affecting fertility
36. Partner’s sperm count	Sperm count of the patient’s partner
37. Partner’s sperm motility	Sperm motility of the partner
38. Partner’s sperm morphology	Morphology of the partner’s sperm
39. Ovarian reserve tests	Ovarian reserve test results
40. Genetic history	Family history of genetic conditions affecting fertility

All the features included in this study were meticulously selected based on expert opinion, ensuring that the chosen attributes are clinically relevant and possess intrinsic value for the prediction of IVF success rates based on live birth occurrences. This expert-driven feature selection process enhances the robustness and clinical applicability of our predictive models.

### 3.2 Preprocessing

In the domain of data preprocessing, a distinctive technique was employed to tackle the issue of null values within the dataset, constituting a notable contribution of this study. Rather than opting for outright removal, a judicious strategy was pursued, involving the replacement of null entries with specific numerical placeholders same as (Jerez et al., 2010). This innovative approach served a dual purpose: firstly, to retain all cases within the dataset, including those with missing feature values, to preserve potentially valuable information; and secondly, to prevent inadvertent escalation of correlations or similarities between cases arising from the presence of null features. By uniquely assigning numerical identifiers to distinct missing values within each feature, the potential for confounding patterns to emerge from shared null entries was effectively mitigated.

Notably, this technique of encoding missing values emerged as a substantial methodological contribution to the study. Its implementation showcased a profound awareness of the challenges associated with missing data, met with an inventive solution. Moreover, the technique played a pivotal role in controlling the rise of similarity between samples, thus safeguarding the robustness and dependability of subsequent classification analyses. The deliberate inclusion of this technique reinforced the study’s overarching objective of generating accurate and trustworthy results within the context of machine learning-based modeling. In essence, this encoding technique underscored the study’s dedication to methodological rigor and empirical validity, thereby augmenting the scholarly value of the research outcomes.

## 4 Methodology

Our methodology involved several critical steps to ensure robust model performance. First, we conducted comprehensive data preprocessing, including handling missing data using numerical placeholders, normalizing numerical features using a Standard Scaler, and encoding categorical variables (e.g., patient ethnicity, treatment type) using one-hot encoding. These steps were crucial for preparing the data for machine learning models.

Next, we applied feature selection guided by domain experts, reducing the dataset to 40 key clinically relevant features, such as patient age, number of embryos transferred, and infertility causes. These features were selected for their potential impact on IVF outcomes.

For model selection and training, we compared a range of machine learning algorithms, including Logistic Regression, SVM, MLP, k-NN, and ensemble methods like AdaBoost and LogitBoost. An 80/20 train-test split was used for training and validation, along with 10-fold cross-validation to ensure model robustness.

Model evaluation was performed using multiple metrics, including accuracy, precision, recall, F1-score, and ROC-AUC, to comprehensively assess the models’ performance. The ensemble models (AdaBoost, LogitBoost) demonstrated superior performance, achieving an accuracy of 96.35%. These additional aspects of our methodology demonstrate that our approach goes beyond hyperparameter tuning and encompasses a holistic machine learning pipeline designed to optimize IVF outcome prediction.

For the purpose of predicting IVF success rates predicated upon the occurrence of live births, our study employed an assortment of machine learning models, each tailored to address specific aspects of the prediction task. The ensemble of models encompassed the Random Forest, Logistic Regression, Gaussian Naive Bayes, Support Vector Machine (SVM) classifier with an RBF kernel, k-Nearest Neighbors (k-NN) classifier, and Multi-layer Perceptron (MLP) classifier. Prior to model assessment, our dataset underwent an 80/20 train-test split, where 20% of the data was reserved for testing, striking a balance between model evaluation and performance validation. To ensure reproducibility and comparability across iterations, we set the random state to a fixed value of 42.

Commencing our analysis with the Random Forest model, we leveraged its ensemble learning approach, amalgamating numerous decision trees to bolster prediction accuracy while curbing overfitting tendencies. Notably, this ensemble was instantiated with 100 decision trees, each contributing to the collective prediction. The random state of 42 in this context guaranteed consistent results, as its value remained unaltered across various model executions.

Segueing into Logistic Regression, a simpler yet interpretable model, we tackled the task of convergence by setting the maximum iteration limit at 10,000. The random state, also established as 42, further fortified the reproducibility of our findings. Logistic Regression offered a transparent lens through which the relationships between features and the likelihood of IVF success could be discerned, facilitating an intuitive understanding of the influencing factors.

Next, Gaussian Naive Bayes was harnessed, capitalizing on its assumption of feature independence for efficient probability estimation. By harnessing prior probabilities and likelihoods, this probabilistic model adeptly inferred class labels, furnishing insights into the prediction of IVF success rates. The Linear SVM classifier emerged as another integral component of our study. By adopting an RBF kernel and incorporating a regularization parameter (C) set at 10, alongside the hinge loss, we aimed to discern patterns in the prediction task, effectively separating classes. This model proved invaluable in decoding intricate relationships underlying the IVF success prediction based on live birth occurrences.

Turning to the k-NN classifier, an instance-based learning method, we engaged with the proximity of neighboring data points to ascertain class labels. Specifically, we adopted k = 5 neighbors, harnessing localized patterns to inform our predictions. Lastly, the Multi-layer Perceptron (MLP) classifier, a neural network variant, was employed to capture intricate data relationships. Configured with a hidden layer containing 100 units and a maximum iteration cap of 1,000, the model delved deep into data intricacies. The random state of 42 maintained consistency in our findings across the neural network’s learning iterations.

**Figure 1:**
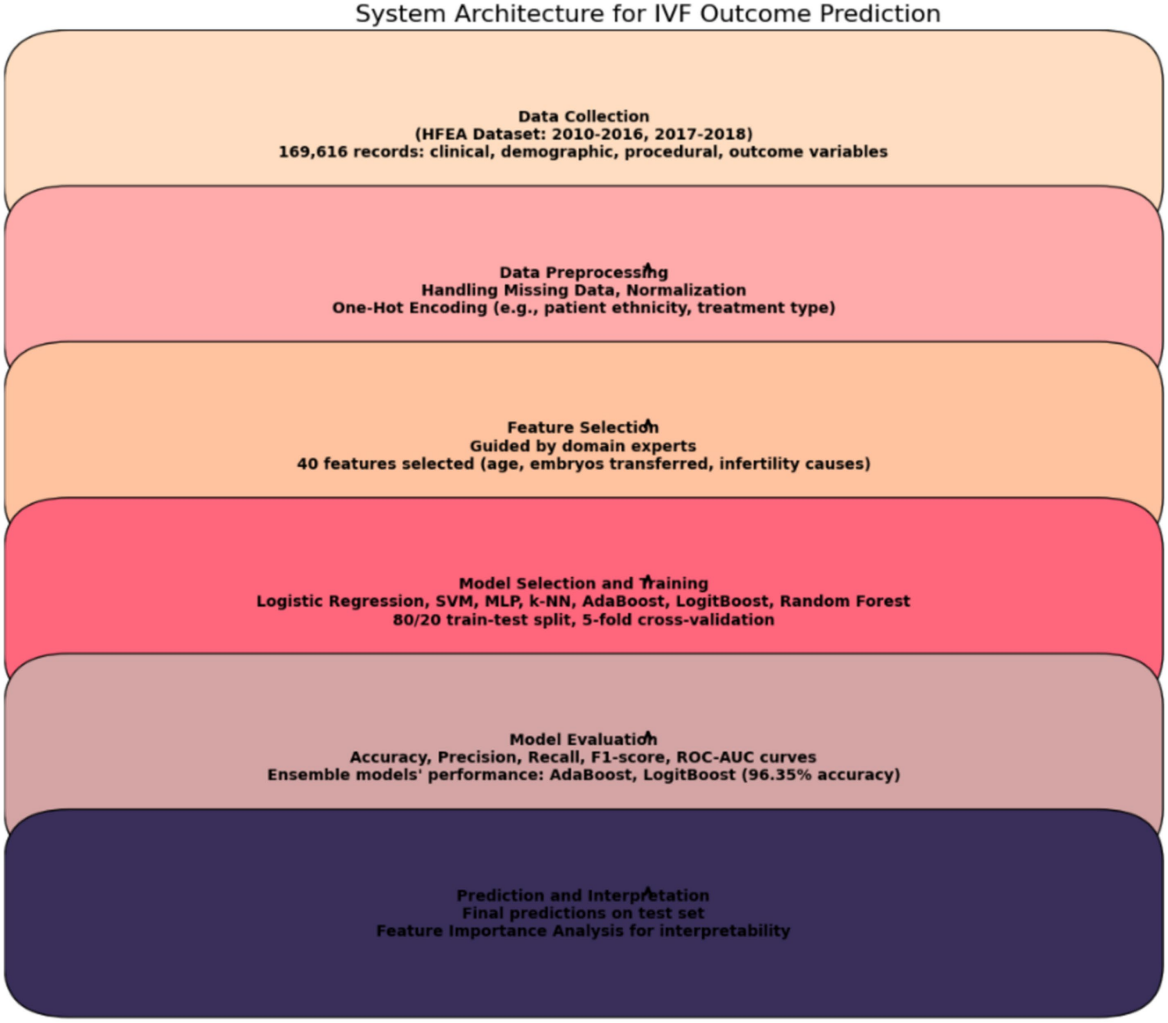
System architecture for IVF outcome prediction.

This concerted application of diverse machine learning models enabled us to delve into the intricate realm of IVF success prediction based on live birth occurrences, thereby enriching our understanding of the underlying factors contributing to this complex medical outcome. Figure 1 illustrates the systematic process for predicting *In vitro* fertilization (IVF) outcomes. The system starts with data collection, which utilizes the HFEA dataset consisting of clinical, demographic, procedural, and outcome variables. It proceeds with data preprocessing, including handling missing data, normalization, and encoding of categorical variables. Next, the feature selection process narrows down the dataset to 40 key features, guided by domain experts. Model selection and training involves a variety of machine learning models, such as Logistic Regression, SVM, and ensemble methods like AdaBoost. Finally, the model evaluation assesses the models using key performance metrics like accuracy and ROC-AUC, followed by prediction and interpretation, where feature importance is analyzed for clinical interpretability.

In the pursuit of predicting IVF success rates based on the occurrence of live births, our methodology extended to encompass Ensemble learning models, which collectively harness the power of multiple base models to enhance predictive performance. Beginning with ‘Adaboost’, an Adaptive Boosting technique, we embraced its inherent capability to progressively refine predictions. By employing 100 weak learners and a deterministic random state of 42, this model iteratively adjusts to give more weight to previously misclassified instances, thereby enhancing the ensemble’s accuracy.

‘GentleBoost’ was another model of choice. Here, we used the DecisionTreeRegressor as the weak learner, with a depth constraint of just one level. GentleBoost’s uniqueness stems from its ability to focus on residuals. Each subsequent model in the ensemble tries to correct the errors of its predecessor, leading to an aggregate model that’s robust against diverse types of errors. Mirroring the iterative ethos of ‘GentleBoost’, the ‘LogitBoost’ model, too, trains on residuals but in a logistic regression framework. This iterative refinement, combined with the logistic aspect, ensures that the ensemble does not just predict but also quantifies the uncertainty of its predictions, offering more nuanced insights into IVF success probabilities.

Diving deeper into ensemble diversity, we explored the ‘RandomSubspaceMethod’ (RSM). Unlike traditional ensembles that rely solely on aggregating different models, RSM introduces variability by using different feature subsets for each model. This ensures that each model in the ensemble views the data from a slightly different angle, making the collective decision more comprehensive and less prone to specific feature biases. Addressing the perennial challenge of class imbalance, we incorporated ‘RusBoost’. This model stands out for its ability to under-sample the majority class, ensuring that the minority class, often of higher interest, is adequately represented and learned from. In a domain like IVF, where success rates can be imbalanced, ‘RusBoost’ ensures that predictions aren’t skewed by mere majority trends but reflect genuine underlying patterns.

Our meticulous integration of Ensemble learning models, spanning the spectrum from ‘Adaboost’ to ‘RusBoost’, has empowered us to extract nuanced insights from the IVF data. Each model, with its unique methodology, has enriched our comprehension of IVF success rate predictions anchored in live birth instances. By tailoring each methodology to its respective nuances, we enhanced our understanding of IVF success rate prediction based on live birth occurrences, contributing to a robust analysis of this intricate medical domain.

To provide a clearer interpretation of the model’s decision-making process, we employed SHAP (Shapley Additive Explanations) to quantify the relative importance of each feature in predicting IVF success. SHAP assigns an importance score to each feature, which represents the contribution of that feature to the model’s final output. This method ensures model transparency and helps identify the most influential clinical parameters that contribute to IVF success prediction.

**Figure 2:**
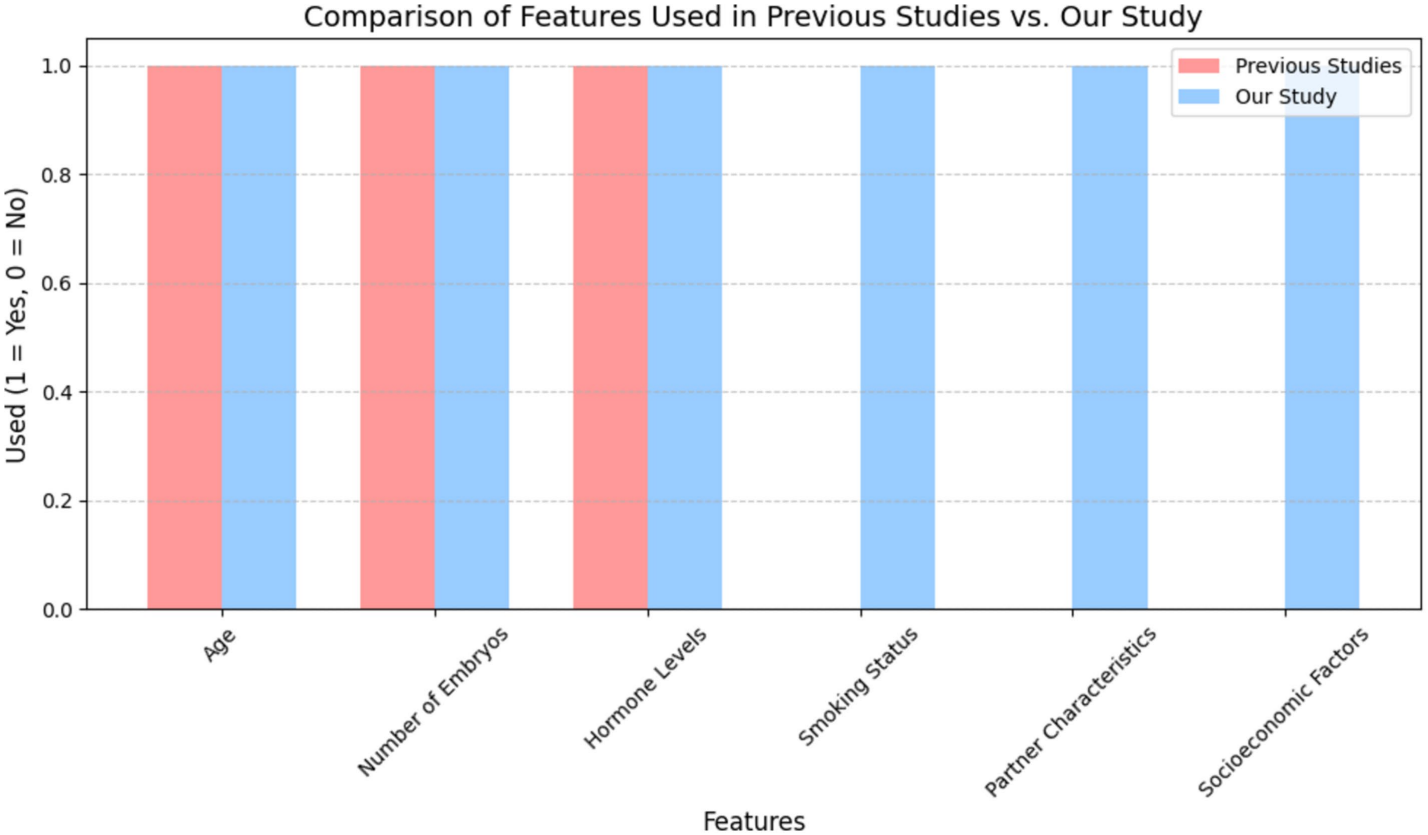
Comparative study of features used in previous IVF prediction models vs. our study. This figure demonstrates that the inclusion of broader lifestyle and partner-related variables, which have been largely underrepresented in earlier studies, improves the model’s predictive performance and clinical applicability.

To justify the integration of a broader spectrum of features, we conducted a comparative analysis of the input features used in previous studies versus those used in this study. As shown in Figure 2, previous models primarily focused on traditional clinical parameters such as patient age, number of embryos transferred, and hormonal profiles. In contrast, our model integrates a wider array of features, including lifestyle, partner characteristics, and socioeconomic factors. This comprehensive feature set allows our model to capture additional dimensions of patient variability that can significantly impact IVF success rates.

## 5 Experiments and results

### 5.1 Data evaluation and statistical analysis

In this study, by analyzing the dataset, it was observed that the factor “number of live births” - our main objective variable - only 24% of the entire group of patients experienced successful IVF outcomes. This factor was binary coded, where “1” indicates successful IVF treatment and “0” indicates failure. Breaking it down, 158,334 patients underwent IVF procedures, with a success rate of 24%. Conversely, of 11,282 patients who underwent DI treatments, 14% reported successful IVF. Demographic analysis showed that a significant proportion of patients, regardless of treatment type, were between 18 and 34 years of age. Specifically, the IVF group had 40% of its patients in this age group, while the DI method reported 50%.

Interestingly, a deeper look at the data revealed that 74% of IVF patients, with no history of live births, had successful treatments. This figure increased to 78% in the DI group. Furthermore, the majority (93%) of successful IVF treatments were performed with patients using their own eggs. This proportion was slightly lower for the DI group, at 90%.

A notable insight from the data set was only three patients showed all enumerated causes of infertility, namely: “tubal disease,” “ovulatory disorder,” “male factor,” “unexplained patient” and “endometriosis.” Despite these multifaceted challenges, these patients achieved successful IVF outcomes. In contrast, 6,676 patients who lacked any of the previously mentioned factors also reported successful IVF outcomes. Table 2 shows a comprehensive analysis of IVF outcomes stratified by specific causes of infertility.

**Table 2:** Detailed perspective on IVF outcomes, segmented by specific infertility causes.

Infertility reasons	Number of success IVF	Number of failed IVF
Causes of infertility - tubal disease	4,094	10,830
Causes of infertility - ovulatory disorder	5,131	12,738
Causes of infertility - male factor	14,039	35,498
Causes of infertility - patient unexplained	11,805	34,457
Causes of infertility - endometriosis	2,328	6,479

Table 3 offers a detailed breakdown, presenting the number of success and fail IVF outcomes across various age brackets, further segmented by the underlying causes of infertility. For each age group, starting from 18 to 34 and ending at 45–50, the table lists the occurrence of live births. It then breaks down the causes of infertility into tubal disease, ovulatory disorder, male factor, unexplained causes, and endometriosis. The data shows a general trend where the number of live births decreases with the increasing age of the patient. For instance, in the 18–34 age group, there were 4,344 cases with no live birth due to tubal disease and 6,938 due to ovulatory disorder, but these numbers drop significantly in the 45–50 age group to 154 and 186, respectively. The same downward trend is observable in the other categories of infertility causes.

**Table 3:** Offers a stratified analysis, displaying IVF outcomes by age groups, further segmented by the underlying cause of infertility.

Patient age at treatment	Number of IVF successes by specific causes of infertility S (Success)/F (Fail)				
	Causes of infertility - tubal disease	Causes of infertility - ovulatory disorder	Causes of infertility - male factor	Causes of infertility - patient unexplained	Causes of infertility - endometriosis
18–34	2,192 (S)	3,223 (S)	7,914 (S)	5,279 (S)	1,246 (S)
	4,344 (F)	6,938 (F)	15,318 (F)	11,155 (F)	2,428 (F)
35–37	1,053 (S)	1,134 (S)	3,310 (S)	3,255 (S)	605 (S)
	2,726 (F)	2,666 (F)	8,234 (F)	8,184 (F)	1,608 (F)
38–39	466 (S)	447 (S)	1,588 (S)	1710 (S)	285 (S)
	1,685 (F)	1,429 (F)	5,201 (F)	6,174 (F)	1,087 (F)
40–42	320 (S)	253 (S)	912 (S)	1,256 (S)	154 (S)
	1,560 (F)	1,211 (F)	4,789 (F)	6,313 (F)	1,058 (F)
43–44	41 (S)	31(S)	163 (S)	194 (S)	21 (S)
	361 (F)	308 (F)	1,285 (F)	1848 (F)	220 (F)
45–50	154 (F)	43 (S)	152 (S)	111 (S)	17 (S)
	22 (S)	186 (F)	671 (F)	783 (F)	80 (F)

During the data pre-processing stage, distinct placeholders were used for absent data to reduce the risk of excessive similarity and correlation among variables. In line with the research framework of this paper and the chosen machine-learning model, non-relevant factors were carefully removed. The refined dataset, therefore, consisted of 36 pivotal factors, consistently analyzed for all patients.

**Figure 3:**
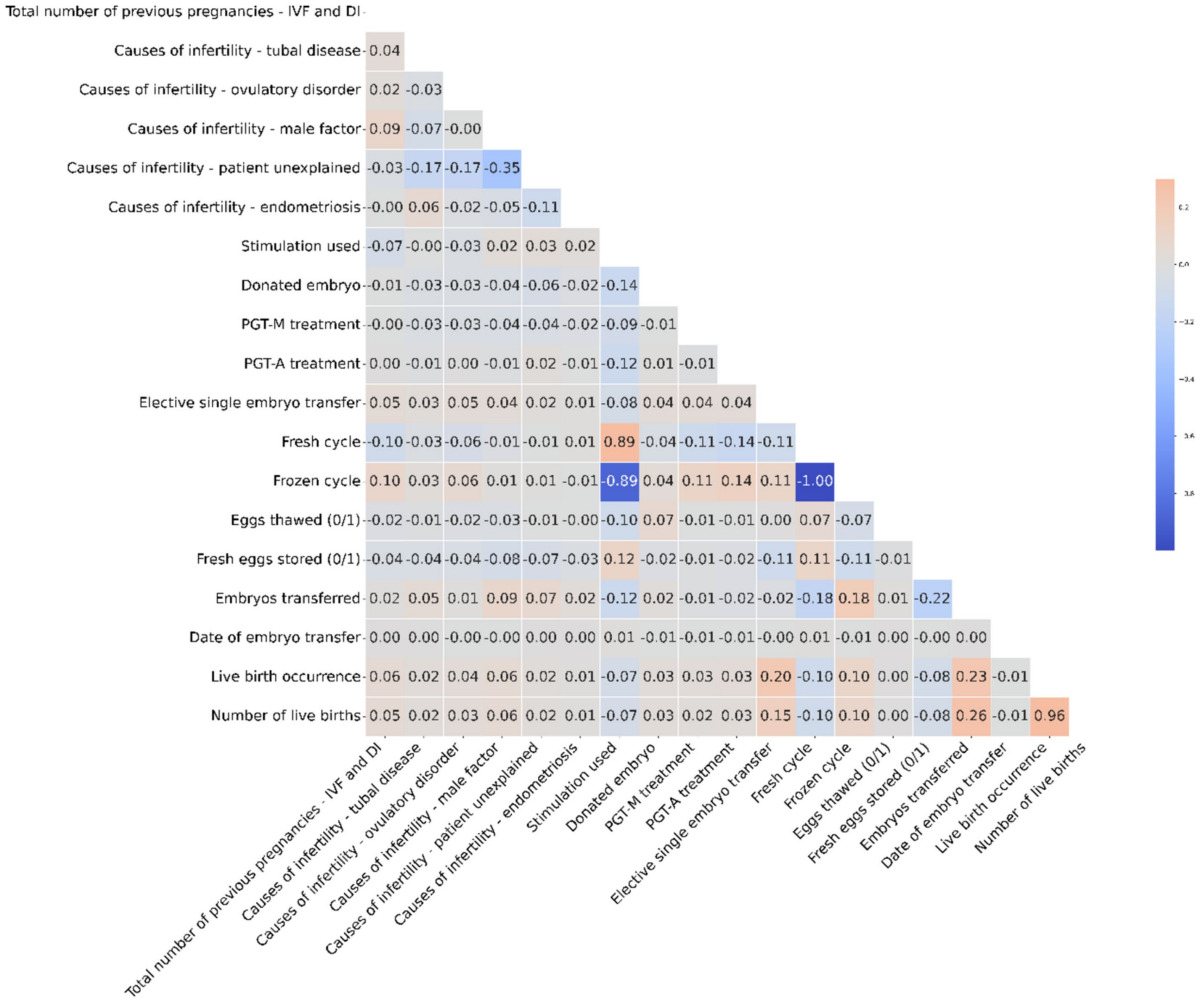
The heatmap illustrates correlations in the IVF dataset, showing strong mutual exclusivity between Fresh and Frozen cycles, while emphasizing that correlation does not necessarily mean causation.

The heatmap visualizes the correlation between numerical columns in the IVF dataset. Red shades signify positive correlations, while blue shades represent negative ones. For instance, Fresh cycle and Frozen cycle have a strong negative correlation of −1, indicating they are mutually exclusive in the dataset. Most other variables show weak linear relationships, with correlation values close to zero. It’s essential to note that correlation does not equate to causation; it merely highlights potential relationships between variables. Figure 3 is a visual representation of correlation coefficients between various factors involved in IVF and DI treatments. A key observation from this heatmap is the strong negative correlation (indicated by a coefficient of −1) between ‘Fresh cycle’ and ‘Frozen cycle’, suggesting that these two variables are mutually exclusive—when one is utilized, the other is not. This is consistent with IVF procedures where typically either a fresh cycle is used, wherein eggs are retrieved and fertilized, and the embryo is transferred in the same cycle, or a frozen cycle is used, where embryos are frozen for later use after a fresh cycle. It is important to highlight that correlation does not imply causation; thus, while these factors are related, we cannot infer that one directly causes the other without further context. Additionally, factors such as ‘Live birth occurrence’ and ‘Number of live births’ show a positive correlation with ‘Eggs thawed’ and ‘Embryos transferred’, indicating that higher numbers in these categories could be associated with successful outcomes, though the correlation is not perfect. This suggests that while the quantity of eggs and embryos plays a role in successful IVF treatments, it is not the sole determinant of success, as quality and other factors can also significantly influence outcomes.

### 5.2 Model evaluation and analysis

Our analysis was rooted in Python, a premier language for data science, enhanced by key libraries like Pandas for data handling and Scikit-learn for a wide array of data mining activities, from preprocessing to modeling and assessment. At the outset, we meticulously segregated the dataset into training and testing segments. This division not only gave a comprehensive overview of the data but also enabled us to train models on one segment and validate them on another, revealing their potential real-world performance.

In the subsequent preprocessing stage, we optimized the dataset for machine learning applications. We addressed missing values, a recurrent challenge in model training, using strategies tailored to each feature. To achieve better and more logical results, we balanced the dataset, ensuring our models would not be biased. We also took measures to ensure that features were as independent as possible, a crucial step to minimize redundancy and enhance the dataset’s quality.

We evaluated the performance of our machine learning models through diverse metrics, each offering a unique insight into their capabilities, such as accuracy, recall, precision, and f1-score (Sadegh-Zadeh, 2019).

Accuracy: Represents the ratio of correct predictions over the total predictions, calculated as Equation 1:


(1) $$\mathrm{Accuracy}=\frac{\mathrm{Number}\;\mathrm{of}\;\mathrm{Correct}\;\mathrm{Predictions}}{\mathrm{Total}\;\mathrm{Predictions}}$$

Precision: Captures the accuracy of positive predictions, defined as Equation 2:


(2) $$\mathrm{Precision}=\frac{\mathrm{True}\;\mathrm{Positives}}{\mathrm{True}\;\mathrm{Positives}+\mathrm{False}\;\mathrm{Positives}}$$

Recall (Sensitivity): Reflects the model’s prowess in identifying all actual positives, given by Equation 3:


(3) $$\mathrm{Recal}=\frac{\mathrm{True}\;\mathrm{Positives}}{\mathrm{True}\;\mathrm{Positives}+\mathrm{False}\;\mathrm{Negatives}}$$

F1-score: A balanced metric that considers both precision and recall, computed as Equation 4:


(4) $$F1=2\times \frac{\mathrm{Precision}\times \mathrm{Recall}}{\mathrm{Precision}+\mathrm{Recall}}$$

**Figure 4:**
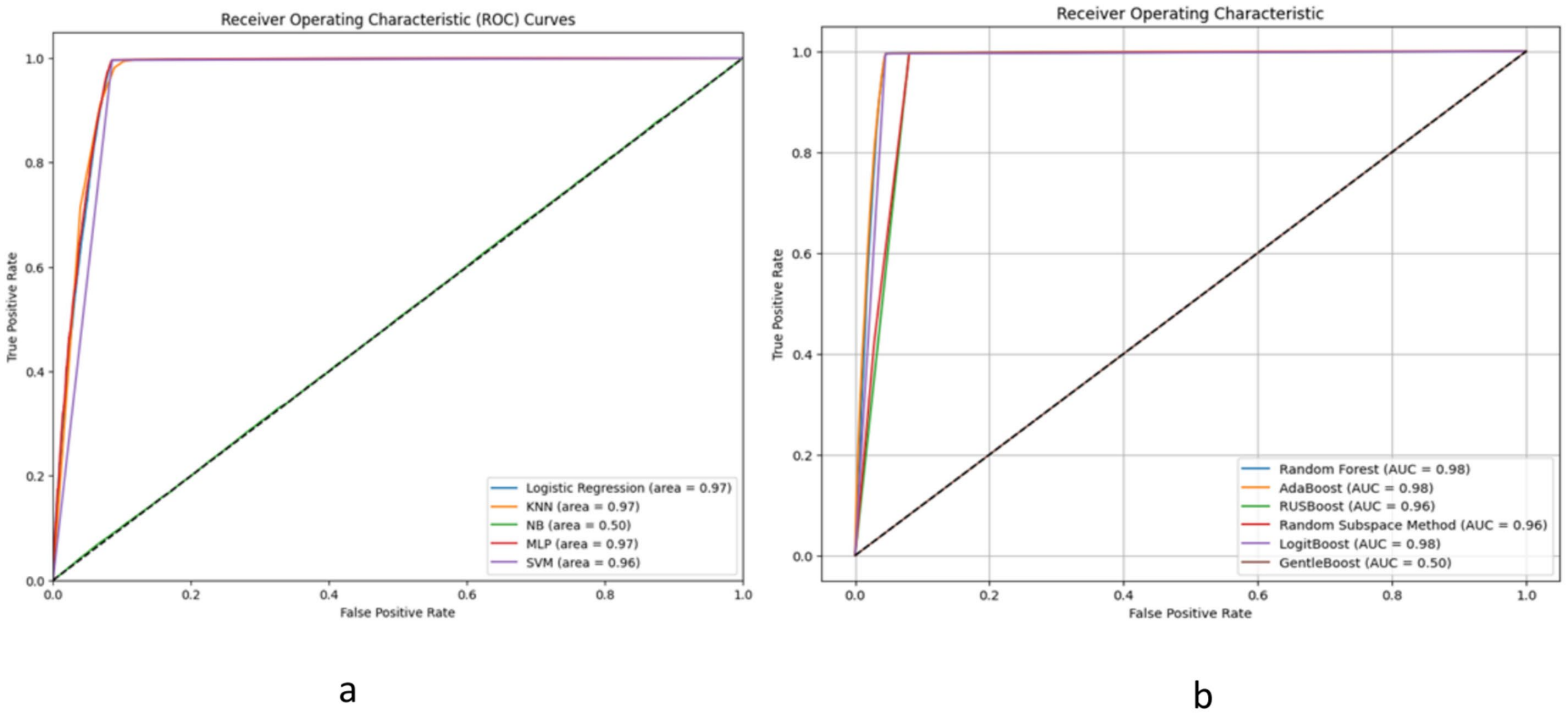
The ROC curve for ML models on HFEA (2010_2016).

Figure 4, alongside Tables 4, 5, provide comprehensive insights into the performance metrics of various machine learning and ensemble learning models, leveraging the HFEA datasets from two distinct periods: 2010–2016 and 2017–2018. In Figure 3A, Logistic Regression, KNN, MLP, and SVM demonstrate high Area Under the Curve (AUC) values close to 0.97, indicating excellent classification performance. In contrast, the Naive Bayes (NB) model shows a significantly lower AUC of 0.50, which is no better than random guessing. Figure 3B includes ensemble methods like Random Forest and AdaBoost, along with other boosting methods such as RUSBoost, Random Subspace Method, LogitBoost, and GentleBoost. Here, most models perform exceptionally well, with AUC values ranging from 0.96 to 0.98, except for GentleBoost which is at 0.50, similar to Naive Bayes in the left graph. High AUC values suggest that these models have a good measure of separability and are able to distinguish between the positive and negative classes effectively. In both graphs, the models with AUC values significantly greater than 0.50 show that they have a good trade-off between sensitivity (true positive rate) and specificity (1 - false positive rate). Models with an AUC of 0.50, represented by the diagonal line, indicate an inability to distinguish between the classes better than random chance. Overall, except for NB and GentleBoost, the models evaluated on the ROC curves demonstrate strong predictive capabilities for the dataset from HFEA (2010–2016).

**Table 4:** Comparison between classification metrics of different models with feature selection on HFEA (2010–2016).

	Model	Accuracy	Precision	Recall	F1-Score	AUC
Machine learning models	Logistic regression	93.25	78.47	99.45	87.72	97
	Gaussian NB	87.30	67.52	91.71	77.78	50
	MLP(epochs = 1,000)	93.35	78.83	99.19	87.86	97
	SVM	93.33	78.66	99.50	87.86	96
	KNN	92.54	77.77	96.94	86.30	97
Ensemble Learning models	AdaBoost	96.35	87.29	99.41	92.96	98
	LogitBoost	96.35	87.29	99.45	92.97	98
	RUSBoost	93.78	79.82	99.50	88.58	96
	Random forest	96.08	87.26	98.15	92.38	98
	RSM	56.75	35.90	99.86	52.81	96

**Table 5:** neural network model hyperparameters.

Parameter	Value
Hidden layers	3
Neurons per layer	64
Activation function	ReLU
Optimizer	Adam
Learning rate	0.001
Epochs	100
Batch size	32
Loss function	Binary crossentropy

The hyperparameters in Table 5 describe the architecture and training configuration of the deep learning model. The model consists of 3 hidden layers, each with 64 neurons, using the ReLU activation function to introduce non-linearity. The Adam optimizer, with a learning rate of 0.001, is employed to adjust the weights during training, while binary crossentropy is used as the loss function for this binary classification task. The model is trained for 100 epochs with a batch size of 32, balancing computational efficiency and model performance. These hyperparameters are crucial for controlling the model’s learning process and generalization ability.

## 6 Comparative analysis and clinical implications

In this section, we provide an in-depth analysis of the comparative performance of various machine learning models employed in our study, discuss the clinical implications of our findings, and suggest pathways for future improvements. The chapter focuses on how these results can be applied in clinical decision-making for improving IVF treatment success rates, highlighting the strengths and weaknesses of the models and offering insights into their applicability in real-world scenarios.

### 6.1 Comparative performance of machine learning models

Our study tested a variety of machine learning models, including traditional classifiers (e.g., Logistic Regression, SVM, k-NN) and ensemble methods (e.g., AdaBoost, LogitBoost, Random Forest), across two datasets (2010–2016 and 2017–2018). Here, we analyze the performance of these models based on key metrics such as accuracy, precision, recall, and F1-score, as well as their clinical relevance.

- *Logistic regression*: Logistic Regression performed relatively well on the dataset, showing an accuracy of 93.25% for the 2010–2016 dataset and 76.63% for the 2017–2018 dataset. However, despite its interpretability and ease of use, Logistic Regression struggled with the complexity of the IVF data, which involves non-linear interactions between variables. LR demonstrated good precision but lower recall, indicating that while it correctly identified many successful IVF outcomes, it missed several positive cases (false negatives), which is a limitation in a clinical setting where minimizing missed successful cases is critical.
- *Support vector machine*: The SVM classifier also showed competitive performance, achieving accuracy rates of 85.77% (2017–2018) and 93.33% (2010–2016). The model’s strength lies in its ability to handle high-dimensional spaces, making it suitable for IVF datasets with complex interactions. However, SVM is computationally expensive and less interpretable than simpler models, which limits its clinical application unless used alongside other tools that can explain its decisions.
- *k-nearest neighbors*: k-NN, while intuitive and simple, did not perform as well as the other models. With accuracy rates of 83.40% (2017–2018) and 92.54% (2010–2016), k-NN’s performance lagged behind more sophisticated models. Its reliance on distance-based calculations made it vulnerable to noisy data and feature scaling issues. Despite these limitations, k-NN can still be useful in specific cases where model simplicity is a priority.
- *Multi-layer perceptron*: MLP, a type of neural network, achieved impressive results, particularly for the 2017–2018 dataset, with an accuracy of 92.18%. MLP is well-suited for capturing non-linear patterns and interactions between features. However, neural networks are typically regarded as black-box models, which presents a challenge in terms of interpretability for clinicians. MLP’s high recall indicates that it is effective at identifying successful IVF outcomes, making it a strong candidate for use in clinical settings when interpretability is less of a concern.
- *Ensemble methods (AdaBoost, LogitBoost, and Random forest)*: The ensemble methods AdaBoost and LogitBoost showed the best overall performance, achieving an accuracy of 96.35% on both datasets. These methods excelled in integrating diverse features (clinical, demographic, and procedural) to provide robust predictions. Their ability to reduce overfitting by combining multiple weak learners makes them highly effective, particularly in handling the high-dimensional, noisy nature of IVF datasets. The use of boosting techniques, which iteratively focus on misclassified instances, was especially valuable in our study, ensuring high recall rates and reducing false negatives. Random Forest, with its built-in feature selection and robustness to overfitting, also performed exceptionally well, achieving a similar accuracy to AdaBoost and LogitBoost.

The impact of removing individual causes of infertility on the best-performing model (Adaboost) was assessed by evaluating key metrics such as accuracy, precision, recall, and F1 score (Table 6). Removing each factor individually led to a slight reduction in model performance, with “Male Factor Infertility” contributing the most to overall accuracy (0.84) and F1 score (0.80). In contrast, removing “Endometriosis” resulted in the lowest recall (0.78) and F1 score (0.77), suggesting that this factor is particularly important for detecting true positive IVF outcomes.

**Table 6:** Impact of removing individual infertility causes on model metrics (2017–2018).

Removing each cause of infertility individually while keeping the others	ACC	Precision	Recall	F1
Tubal disease	0.81	0.76	0.80	0.78
Ovulatory disorder	0.83	0.79	0.80	0.79
Male factor infertility	0.84	0.79	0.81	0.80
Patient unexplained infertility	0.83	0.78	0.80	0.79
Endometriosis infertility	0.81	0.76	0.78	0.77

Comparing model performance with and without all infertility causes highlights the critical role these variables play in improving prediction accuracy (Table 7). Without considering infertility causes, the model achieves an accuracy of 0.86 and an F1 score of 0.84. However, when these factors are included, accuracy and F1 score rise significantly to 0.95 and 0.92, respectively. This improvement, particularly in recall (0.99 with all factors included), demonstrates the substantial influence specific infertility factors have on predicting successful IVF outcomes.

**Table 7:** Comparison of model metrics with and without infertility causes (2017–2018).

Metrics	Without causes of infertility	With causes of infertility
ACC	0.86	0.95
Precision	0.80	0.85
Recall	0.88	0.99
F1	0.84	0.92

The results from the 2010–2016 dataset are consistent with those from 2017 to 2018, demonstrating stable performance when individual infertility factors are removed (Table 8). The model’s accuracy remains within 0.88 to 0.90, with minimal changes in precision, which hovers around 0.81. Removing factors such as “Tubal Disease” and “Male Factor Infertility” results in high recall (0.97), indicating that these factors significantly contribute to the model’s ability to detect true positive outcomes. However, no single cause of infertility shows a substantial impact on the overall F1 score, which remains around 0.88 for most conditions.

**Table 8:** Impact of removing individual infertility causes on model metrics (2010–2016).

Removing each cause of infertility individually while keeping the others	ACC	Precision	Recall	F1
Tubal disease	0.90	0.81	0.97	0.88
Ovulatory disorder	0.89	0.82	0.96	0.88
Male factor infertility	0.89	0.81	0.97	0.88
Patient unexplained infertility	0.88	0.80	0.94	0.86
Endometriosis infertility	0.89	0.81	0.96	0.88

Including all infertility causes in the model results in noticeable performance improvements (Table 9). Accuracy increases from 0.89 to 0.96, precision from 0.81 to 0.87, and the F1 score from 0.89 to 0.92. While recall remains high (0.98) even without these factors, including infertility causes leads to a more balanced and robust model, particularly enhancing precision and overall predictive power. This highlights the importance of incorporating these factors to improve IVF success prediction.

**Table 9:** Comparison of model metrics with and without infertility causes (2010–2016).

Metrics	Without causes of infertility	With causes of infertility
ACC	0.89	0.96
Precision	0.81	0.87
Recall	0.98	0.99
F1	0.89	0.92

Our study presents a comprehensive IVF outcome prediction model that incorporates a broader range of features and a larger dataset compared to previous work. For instance, Hassan et al. (2020) achieved an accuracy of 98.38% using SVM on a dataset of 1,729 records with a feature set of 17–19 variables. In contrast, our model uses a significantly larger dataset and a broader feature set that includes clinical, demographic, and procedural factors, offering more personalized predictions. Additionally, while our highest reported accuracy is 96.35% using ensemble methods like AdaBoost and LogitBoost, these models offer greater stability and interpretability in clinical contexts, which is crucial for decision-making. Furthermore, our evaluation includes a wider range of metrics (precision, recall, F1-score, and ROC-AUC) to ensure comprehensive model performance analysis beyond just accuracy.

### 6.2 Model interpretation

The SHAP analysis revealed that maternal age, previous IVF cycles, and hormone levels (e.g., luteinizing hormone and gonadotropin) were the most important features in predicting IVF success. Table 10 presents the ranked importance of each feature, showing that maternal age had the highest influence, followed by the number of previous IVF cycles and hormone levels. These findings align with clinical expectations, reinforcing the model’s relevance for clinical use.

**Table 10:** SHAP feature importance scores.

Feature	SHAP importance score
Maternal age	0.245
Previous IVF cycles	0.198
Luteinizing hormone	0.153
Gonadotropin dosage	0.142
Infertility type	0.125
Embryo count	0.110
Patient ethnicity	0.075
Partner ethnicity	0.062
Number of embryos transferred	0.052

### 6.3 Clinical relevance and implications

The high predictive accuracy of the ensemble models, particularly AdaBoost and LogitBoost, demonstrates their suitability for clinical use in predicting IVF success. However, predictive performance alone is not enough to justify their use in real-world IVF clinics. The interpretability, clinical relevance, and scalability of these models are also key factors.

1. *Personalized treatment planning*: By accurately predicting the likelihood of IVF success based on a comprehensive set of clinical and demographic factors, these models can help healthcare providers tailor treatment protocols to individual patients. For example, patients with lower predicted success rates may benefit from more aggressive or alternative treatments, while those with higher predicted success rates could be counseled to continue with standard protocols. The inclusion of detailed clinical parameters (e.g., hormone levels, embryo quality, and patient demographics) in the models ensures that treatment plans are informed by a holistic understanding of each patient’s reproductive profile.
2. *Decision support systems*: The strong performance of ensemble models makes them excellent candidates for integration into clinical decision support systems (CDSS). These systems could provide real-time predictive insights based on patient data, assisting clinicians in making evidence-based decisions. For instance, AdaBoost and LogitBoost could be integrated into electronic medical records (EMRs) to provide automatic predictions of IVF success when new patient data is entered. The recall rates of these models suggest they are particularly effective at identifying successful cases, which is crucial for advising patients about their chances of success.
3. *Reducing emotional and financial stress*: IVF treatment is emotionally and financially taxing. Providing patients with reliable success predictions can help them make more informed decisions about whether to pursue additional cycles or explore alternative options. The high accuracy of our models offers patients and clinicians a level of confidence in the predictions, reducing the uncertainty and stress associated with IVF treatments.
4. *Handling of missing data*: One of the key innovations in our approach is the novel handling of missing data, where missing entries were replaced with numerical placeholders. This method preserved the integrity of the dataset and ensured that important cases were not discarded due to incomplete data. Clinically, this is significant because real-world datasets often have missing values, and our model’s ability to effectively manage this limitation ensures that predictions remain robust and applicable to a wider patient population.
5. *Interpretability and clinical adoption*: Despite the strong performance of ensemble methods, the complexity of these models can be a barrier to clinical adoption. To address this, we focused on making the models as interpretable as possible. For example, by providing explanations of which features (e.g., age, previous IVF cycles, and hormone levels) contributed most to the prediction, clinicians can better understand and trust the model’s output. In future developments, tools like SHAP (Shapley Additive Explanations) could be integrated to enhance model transparency and clinician trust further.

### 6.4 Future clinical applications

Looking forward, the use of AI and machine learning in reproductive medicine offers numerous potential benefits:

- *Real-time monitoring*: Machine learning models could be integrated into continuous monitoring systems that track patient progress throughout the IVF process, providing real-time feedback and adjusting treatment protocols dynamically based on evolving patient data.
- *Predictive maintenance of lab equipment*: Machine learning could also be applied to predict and prevent equipment failures in IVF labs, ensuring that the highly sensitive processes involved in embryo fertilization and transfer are not disrupted by unexpected technical issues.
- *Integration with wearable devices*: The use of wearable health devices that track physiological parameters (e.g., heart rate, body temperature, stress levels) could feed additional data into the predictive models, allowing for even more personalized treatment planning.

## 7 Discussion

The results presented in this study represent a significant step forward in the application of advanced machine learning paradigms to predict *In-Vitro* Fertilization (IVF) success rates. The study utilized two different datasets, one from 2017 to 2018 and another from 2010 to 2016, to explore the effectiveness of various machine learning models in predicting live-birth occurrences in IVF cycles. In Figure 3, the ROC curves for machine learning models evaluated on the HFEA dataset (2010–2016) are presented. Notably, the ensemble models, such as AdaBoost and Random Forest, exhibited strong performance with AUC values ranging from 0.96 to 0.98, indicating excellent classification ability. The detailed comparison of model performance can be found in Table 11 for the 2017–2018 dataset and Table 4 for the 2010–2016 dataset. The AdaBoost and LogitBoost models, in particular, consistently achieved high accuracy (96.35%) across both datasets, as outlined in these tables, reinforcing their robustness and suitability for IVF outcome prediction. The findings shed light on both the machine learning and gynecological aspects of IVF success prediction. The research objectives revolved around exploring advanced machine learning models for IVF success prediction. The study effectively addressed these objectives by comparing various machine learning algorithms, including neural network, across two different datasets. It also considered the gynecological factors that contribute to IVF success, providing a holistic perspective on the problem.

**Table 11:** Comparison between classification metrics of different models with feature selection on HFEA (2017–2018).

	Model	Accuracy	Precision	Recall	F1-Score	AUC
Machine learning models	Logistic regression	76.63	57.84	63.62	60.59	88
	Gaussian NB	75.63	53.65	7.16	12.63	49
	MLP(epochs = 1,000)	92.18	77.11	97.02	85.93	97
	SVM	85.77	63.49	99.27	77.45	90
	KNN	83.40	63.11	78.34	69.91	93
Ensemble Learning models	AdaBoost	95.78	85.75	99.38	92.06	98
	LogitBoost	95.78	85.75	99.38	92.06	97
	RUSBoost	75.25	49.85	99.40	66.40	83
	Random forest	95.13	85.68	96.34	90.70	98
	RSM	47.71	31.98	99.90	48.46	97

### 7.1 Gynecological implications

- Patient demographics: The study’s results suggest that certain patient demographics and characteristics play a significant role in IVF success. Factors such as maternal age, years of infertility, gonadotropin (Gn) dosage, luteinizing hormone (LH) levels, and ovarian response are all associated with different success rates. These findings align with gynecological knowledge that these factors impact fertility outcomes (Huang et al., 2019; Yu et al., 2019).
- Infertility factors: The analysis revealed that infertility factors, such as male factor, ovulatory dysfunction, and tubal factor, were associated with better live birth rates, while patients with a poor ovarian response had lower odds of success. These findings underscore the importance of considering the underlying causes of infertility when predicting IVF outcomes, which is consistent with gynecological principles (Carson and Kallen, 2021).
- Oocyte activation and fertilization: Notably, patients with low fertilization rates in the first cycle showed higher live birth rates when oocyte activation agents were used in subsequent cycles. This observation reinforces the clinical significance of oocyte activation techniques and their potential to improve IVF outcomes, aligning with gynecological practices (Bonte et al., 2019).
- Factors influencing success: The study identifies several factors that appear to influence the likelihood of a successful IVF outcome in subsequent cycles. These factors include shorter years of infertility, lower gonadotropin (Gn) dosage, and lower luteinizing hormone (LH) levels in the first cycle. This information can guide gynecologists in making informed decisions regarding treatment plans. For instance, patients with these favorable characteristics may require less aggressive stimulation protocols, reducing the risk of overstimulation and its associated complications.
- Relevance of multiple cycles: The study aligns with previous research in suggesting that the majority of patients achieve live births within the first three IVF cycles. This underscores the importance of continuity in treatment. Gynecologists should counsel patients about the potential need for multiple cycles to increase their chances of success, especially when dealing with certain infertility factors.

The parameter dependencies in our analysis were identified using both machine learning model outputs and a heat map analysis. The machine learning models, through feature importance evaluation and SHAP (Shapley Additive Explanations) values, highlighted the significant features influencing IVF success. These insights were further supported by a heat map, which illustrated the correlations between clinical variables such as patient age, hormone levels, infertility type, and previous IVF attempts. This dual approach ensures that the dependencies observed are both technically robust and clinically meaningful. For example, infertility factors like male factor, ovulatory dysfunction, and tubal factor are associated with better live birth rates due to their treatable nature. Clinically, interventions for male factor infertility (e.g., intracytoplasmic sperm injection) and ovulatory dysfunction (e.g., ovulation induction) often lead to higher success rates. This aligns with the results shown in Table 2, where male factor infertility led to a higher number of successful IVF outcomes compared to unexplained infertility. In contrast, patients with a poor ovarian response generally experience lower success rates because their ovarian reserve is diminished, reducing the number of eggs available for fertilization. This is supported by clinical research and our findings in Table 3, where patients with poor ovarian response consistently had lower success rates across all age groups. The machine learning models also identified ovarian response as a key predictor, with poor response significantly lowering the predicted probability of success.

### 7.2 Healthcare practitioners and patients implications

- Clinical decision support: Healthcare practitioners involved in reproductive medicine can benefit from the study’s findings by incorporating machine learning models into their practice. These models can serve as valuable decision support tools, helping clinicians provide more accurate prognostications to IVF patients.
- Patient counseling: Patients considering IVF can gain insights into their potential success rates based on their demographic and infertility factors. This information can guide informed decision-making and help manage patient expectations.

### 7.3 Machine learning implications

Data Preprocessing: The study’s approach to data preprocessing, including balancing the dataset, one-hot encoding, and feature selection, is crucial for enhancing model performance. These techniques help mitigate biases and reduce the risk of overfitting. Gynecologists and data scientists should collaborate to ensure that the data used for predictive modeling is appropriately prepared.

- Model selection: The study employed a variety of machine learning models, including Logistic Regression, Gaussian NB, SVM, MLP, KNN, as well as ensemble learning models like Random Forest, AdaBoost, Logit Boost, RUS Boost, and RSM. Additionally, neural network techniques were applied. These models yielded accuracy rates ranging from approximately 57 to 96%, depending on the dataset and model choice. Such diversity in models provides valuable insights into the best-performing algorithms for IVF success prediction.
- Ensemble learning: Ensemble learning techniques, including AdaBoost and Random Forest, also showed high accuracy. These models can be valuable in clinical decision support systems, providing gynecologists with reliable predictions. However, model interpretability should be considered when using ensemble methods in clinical practice.
- Model performance: The machine learning models employed in this study demonstrated varying degrees of accuracy in predicting IVF success rates. Models such as Random Forest, AdaBoost, and Logit Boost achieved high accuracy, highlighting their potential for clinical application. However, it’s essential to recognize that machine learning models are tools that can aid decision-making but should not replace clinical expertise.
- Neural network results: It achieved accuracy rates of approximately 91.44% for the 2017–2018 dataset and 94.71% for the 2010–2016 dataset. These results indicate the potential of neural network techniques in improving IVF success prediction, as they can capture intricate patterns within the data.

### 7.4 Combined insights

The collaboration between gynecologists and data scientists is pivotal in harnessing the full potential of machine learning for IVF success prediction. Integrating advanced machine learning paradigms into clinical practice can offer several advantages:

- Personalized treatment: Machine learning models can assist gynecologists in tailoring IVF treatment plans based on individual patient profiles. By considering factors like years of infertility, hormone levels, and infertility causes, treatment strategies can be optimized for better outcomes.
- Risk assessment: Machine learning models can provide gynecologists with risk assessments for patients who have experienced initial IVF failure. This information allows for more informed discussions with patients about the likelihood of success in subsequent cycles, helping manage expectations and emotional stress.
- Treatment optimization: The identification of factors influencing IVF success can guide gynecologists in optimizing treatment protocols. For instance, patients with favorable characteristics may benefit from less aggressive stimulation, reducing the risk of complications.
- Continuous monitoring: Machine learning models can be integrated into ongoing patient care to provide real-time predictions and adapt treatment strategies as needed. This ensures that treatment plans remain aligned with each patient’s evolving profile and circumstances.
- Research and collaboration: Gynecologists and data scientists can collaborate on further research to refine predictive models and improve their clinical utility. Larger multicenter studies can validate findings and enhance the generalizability of predictive models.

While several future research opportunities exist, it is important to note that the integration of machine learning models into current clinical practice offers immediate benefits. For instance, machine learning models can be employed to personalize treatment, assist in risk assessments, and optimize treatment strategies based on real-time data. These models provide continuous monitoring of patient progress, allowing for dynamic adjustments in IVF treatment protocols. Additionally, the collaboration between gynecologists and data scientists will further enhance the clinical utility of these models, refining predictive accuracy and improving patient outcomes in the near term. However, further work is required to generalize the models to other datasets and clinical settings, ensuring broader applicability and refinement. Long-term research could focus on external validation, larger multi-center studies, and integrating these models more seamlessly into clinical decision support systems.

### 7.5 Potential limitations of the study

One potential limitation of the study is its retrospective nature, which hinders the ability to effectively control for unknown confounding factors. Prospective studies, incorporating controlled variables, could offer more robust findings by minimizing the impact of unaccounted variables on the results.

### 7.6 Future directions

In the realm of future directions, prospective studies offer a promising avenue for research, enabling the acquisition of meticulously controlled data that facilitates in-depth analyses and the discernment of causal factors. Furthermore, an imperative step involves external validation of the machine learning models devised in this study, employing data from various IVF centers to gage their broader applicability and generalizability. Moreover, there is potential for continued exploration into the seamless integration of these machine learning models within clinical practice, potentially serving as valuable tools to aid clinicians in rendering real-time decisions throughout the course of IVF treatment.

This study represents a significant step toward enhancing IVF success rate prognostication by combining gynecological expertise with advanced machine learning techniques. The findings offer valuable guidance for both gynecologists and data scientists in optimizing IVF treatment plans, managing patient expectations, and improving overall success rates in assisted reproduction. Collaborative efforts in this domain have the potential to revolutionize fertility treatments and provide hope to millions of couples worldwide grappling with infertility challenges.

## 8 Conclusion

This study represents a significant milestone in the field of assisted reproductive medicine, particularly in the context of predicting *In-Vitro* Fertilization (IVF) success rates. By integrating the expertise of gynecologists with advanced machine learning techniques, we have gained valuable insights that hold great promise for both healthcare practitioners and patients alike. From a gynecological perspective, our findings underscore the critical role of patient demographics and infertility factors in IVF success. Maternal age, years of infertility, gonadotropin dosage, luteinizing hormone levels, and ovarian response have all emerged as key determinants of live birth rates. These insights align with established gynecological knowledge, emphasizing the importance of personalized treatment plans that consider these factors. Moreover, our study highlights the significance of addressing underlying infertility causes when predicting IVF outcomes, such as the positive impact of certain infertility factors like male factor, ovulatory dysfunction, and tubal factor on live birth rates. Furthermore, our exploration of oocyte activation and fertilization rates in relation to IVF success provides valuable clinical insights. Patients with low initial fertilization rates who subsequently received oocyte activation agents showed improved live birth rates, affirming the clinical relevance of such techniques. The identification of specific factors influencing success, including shorter years of infertility, lower gonadotropin dosage, and lower luteinizing hormone levels, empowers gynecologists to make more informed treatment decisions tailored to individual patient profiles. From a machine learning perspective, our study showcases the potential of various algorithms and models in predicting IVF success. Machine learning techniques, including ensemble learning and neural network, have demonstrated accuracy rates ranging from 57 to 96%, depending on the dataset and model choice. Notably, neural network techniques have proven effective in capturing intricate patterns within the data, achieving accuracy rates of approximately 91.44 and 94.71% for the two datasets, respectively. These findings highlight the versatility and potential of machine learning in improving IVF success prediction. Our findings offer gynecologists valuable tools for personalized treatment planning, risk assessment, and treatment optimization. For patients, this means more informed decision-making and better-managed expectations during their IVF journey.

## Data Availability

The original contributions presented in the study are included in the article/supplementary material, further inquiries can be directed to the corresponding author.
